# Prevalence, Biogenesis, and Functionality of the Serine Protease Autotransporter EspP

**DOI:** 10.3390/toxins5010025

**Published:** 2012-12-28

**Authors:** André Weiss, Jens Brockmeyer

**Affiliations:** Institute of Food Chemistry, Corrensstraße 45, Münster 48149, Germany; E-Mail: aweis_01@uni-muenster.de

**Keywords:** EspP, EHEC, virulence factor, SPATE, autotransporter, serine protease

## Abstract

Enterohemorrhagic *E*. *coli* (EHEC) causes severe diseases in humans worldwide. One of its virulence factors is EspP, which belongs to the serine protease autotransporters of *Enterobacteriaceae* (SPATE) family. In this review we recapitulate the current data on prevalence, biogenesis, structural properties and functionality. EspP has been used to investigate mechanistic details of autotransport, and recent studies indicate that this transport mechanism is not autonomous but rather dependent on additional factors. Currently, five subtypes have been identified (EspPα-EspPε), with EspPα being associated with highly virulent EHEC serotypes and isolates from patients with severe disease. EspPα has been shown to degrade major proteins of the complement cascade, namely C3 and C5 and probably interferes with hemostasis by cleavage of coagulation factor V. Furthermore, EspPα is believed to contribute to biofilm formation perhaps by polymerization to rope-like structures. Together with the proteolytic activity, EspPα might ameliorate host colonization and interfere with host response.

## 1. Introduction

The plasmid-encoded extracellular serine protease EspP is one of the most abundant proteins in culture supernatants of Shiga-toxin producing *Escherichia coli* (STEC) and enterohemorrhagic *E*. *coli* (EHEC) [1,2] and has been first described on the large plasmid pO157 of EHEC O157:H7 strain EDL933 [1]. The *espP* gene consists of a 3900 bp open reading frame that encodes the 1300 aa EspP protein with a molecular weight of 142 kDa. Cleavage of the *N*-terminal signal peptide and the *C*-terminal β-domain leads to the mature secreted passenger domain. The mature protein found in the extracellular milieu is 104 kDa large and shows serine protease activity [1]. In the same year, Djafari *et al.* described a serine protease of identical size from a bovine *E*. *coli* O26:NM strain and designated the protein PssA (protease secreted by STEC) [3]. Comparative analysis revealed however that EspP and PssA differ by only eight bases on the nucleotide level and a single point mutation on the protein level. PssA is thus an isoform of EspP.

EspP belongs to the family of serine protease autotransporters of *Enterobacteriaceae* (SPATE) [1] and has been used as a prototype to study the autotransport mechanisms. An early definition of this secretion system suggested that autotransporters harbor all components required for translocation through the inner and outer membrane within one entity [4,5,6]. However, various recent studies have demonstrated the interaction with further accessory factors [7,8,9,10,11,12], indicating that autotransport is not independent and might be a misnomer. More than 1500 members of the autotransport family have been identified, probably forming the largest group of proteins secreted by Gram-negative bacteria [13]. Autotransporters show considerable structural and functional differences and include adhesins, proteases and lipases. As indicated by their name, SPATEs exhibit serine protease activity and are secreted by *Enterobacteriaceae*. Although some SPATEs have been found in non-pathogenic bacteria [14] they are clearly associated with pathogenic organisms [6,15,16,17,18]. Table 1 shows selected SPATE proteins from pathogenic *E*. *coli* [19,20].

**Table 1:** Selected serine protease autotransporters of *Enterobacteriaceae* (SPATEs) in pathogenic *E*. *coli*. ETEC, enterotoxigenic *E*. *coli*, EPEC, enteropathogenic *E*. *coli*, APEC, avian pathogenic *E*. *coli*, UPEC, uropathogenic *E*. *coli*, EAEC enteroaggregative *E*. *coli*, DEAC diffusely aggregating *E. coli*.

Protein	Organism	Function/Effects	Reference
EatA (ETEC autotransporter A)	ETEC	Mucosal destruction	[21]
EpeA (EHEC plasmid-encoded autotransporter)	EHEC	Mucinase activity	[22]
EspC (EPEC secreted protein C)	EPEC	Mediation of EPEC lysozyme resistance, vacuolation, cell rounding and detachment, cytoskeletal damage	[23,24,25]
EspI (*E*. *coli* secreted protease, island-encoded)	STEC	Unknown	[26]
EspP/PssA (extracellular serine protease, plasmid-encoded/protein secreted by STEC A)	EHEC	See this review	[1,3]
Hbp/Tsh (hemoglobin protease/temperature-sensitive hemagglutinin)	Human septic *E*. *coli*/APEC/UPEC	Binding of hemoglobin/heme, degradation of hemoglobin, hemagglutinin, adhesion, mucinase activity	[27,28,29,30,31]
Pet (plasmid-encoded toxin)	EAEC	Inflammation, mucus secretion, tissue damage	[32,33]
Pic/PicU (protease involved in intestinal colonization)	EAEC/UPEC	Hemagglutinin, serum resistance mediator, mucinase activity	[34,35]
Sat (secreted autotransporter toxin)	DAEC/EAEC/UPEC	Causes autophagy, vacuolating toxicity, cell detachment and elongation, formation of lesions in tight-junctions	[18,36,37,38,39]
SepA (*Shigella* extracellular protein)	EAEC	Tissue inflammation, mucosal atrophy, fluid accumulation	[40,41]
SigA (*Shigella* IgA-like protease homolog)	EAEC	Cell rounding and detachment	[40,42]
Vat (vacuolating autotransporter toxin)	APEC	Vacuolating toxicity	[43]

In general, SPATE proteins are believed to contribute to the virulence of their bacterial host by the secreted passenger domains which all harbor the serine protease motif GDSGS (with S being the catalytic serine residue). Despite the overall similar fold, it appears that substrate specificity is distinct for the known SPATEs and that these differences at least partially translate into distinct biological functions [23,34,36,44,45,46] (Table 1). 

For EHEC, Shiga toxins are regarded as major virulence factors which are however not sufficient *per se* for high pathogenicity. Additional virulence factors are necessary that mediate bacterial adherence or interfere with the host response [47,48]. This is exemplified by strains that lack LEE-encoded virulence factors and often show mild clinical outcomes probably due to insufficient adherence. However, bacterial adherence can also be mediated by other factors as observed in the European *E*. *coli* O104:H4 outbreak in 2011. In fact, this outbreak suggests that different “recipes” for high virulence do exist for this pathogen [40]. Concerning EspP, there are different subtypes from which the translocation-competent and proteolytically active subtype EspPα is associated with isolates from patients with severe disease [49,50]. EspPα is therefore considered as additional EHEC virulence factor.

## 2. Extracellular Serine Protease Plasmid-Encoded

### 2.1. Organization of EspP

**Figure 1:**
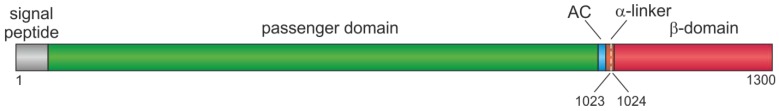
Structural organization of EspP. Signal peptide, amino acids 1–55; passenger domain, amino acids 56–1023; β-domain, amino acids 1024–1300. α-linker, α-helix connecting the passenger domain and the β-domain, amino acids 1014–1028; AC, autochaperone domain which is part of the passenger domain. Autoproteolytic cleavage occurs between Asn^1023^ and Asn^1024^.

EspP consists of a 55 aa *N*-terminal signal peptide (SP), the secreted passenger domain (PD, amino acids 56–1023) and the *C*-terminal β-domain, also called translocator, which is 277 amino acids in length [1] (see Figure 1). This structural organization is seen in all SPATEs. The serine protease motif is located in the PD (see 2.4), while the β-domain is responsible for efficient autotransport through the outer bacterial membrane (see 2.3). The *C*-terminal part of the PD harbors the approximately 30 aa linker-region that connects the β-domain and the PD and is necessary for folding and stability of the β-domain [51,52,53]. In addition, the autochaperone motif (AC) at the passenger *C*-terminus is essential for folding of the β-helical structure of the passenger domain [54,55].

### 2.2. Autotransport

At least seven different pathways exist for the protein transport in Gram-negative bacteria [56]. Autotransport, also referred to as Type V pathway, is probably the most widely distributed mechanism and is divided into classical autotransport (also known as Type Va), the two-partner secretion system (Type Vb), and the trimeric autotransporters (TAA, Type Vc) as well as the two recently described autotransporter Types Vd and Ve [57]. Briefly, the transport through the inner membrane (IM) is mediated by the *N*-terminal signal peptide via the Sec-machinery in the Type V pathway and the *C*-terminal β-domain is inserted into the outer membrane (OM) and facilitates secretion of the passenger domain into the extracellular space. EspP is transported by the Type Va secretion system, and it has to be noted that biogenesis via Type Va secretion apparently shows some diversity in SPATE. We will therefore limit the discussion to novel mechanistic details of EspP biogenesis, which not necessarily fully applies to other SPATE.

**Figure 2:**
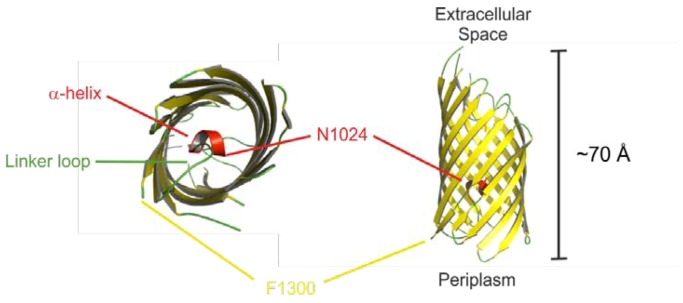
EspP β-domain. The EspP β-domain (residues 1024–1300) forms a 12-stranded β-barrel with a short α-helix and a linker loop at its *N*-terminus. Secondary structure elements are colored in the ribbon diagram: yellow (β-strand), red (α-helix), and green (loop). Some residues (1074–1075, 1135–1137, 1184–1191) are missing in the crystal structure and are therefore omitted [62].

EspP has an unusually long SP which is found in about 10% of autotransporters and which has been shown to facilitate posttranslational targeting [58]. The *C*-terminal part resembles a typical SP with canonical N, H, and C regions (termed N2, H2, and C) while the *N*-terminal part harbors a unique sequence motif (N1 and H1) that is highly conserved among SPATEs from *E*. *coli* and *Shigella* sp. [59,60]. The *N*-terminal extension is not required for efficient translocation across the IM. However, modifications of the SP lead to misfolding of EspP in the periplasm and inhibition of translocation across the OM. The SP transit via the Sec pathway is relatively slow and it is believed that this is due to tethering of the PD to the IM [61] and a slow dissociation from the IM probably due to an unusual conformation of the SP [58]. EspP interacts with several chaperones in the periplasm, namely SurA, Skp, DegP, FkpA, and the Bam complex [7,8,9]. A direct protein-protein interaction of SurA, DegP [8] and FkpA [9] has been observed with the unfolded but not with the native folded PD of EspP, and it has been shown that *surA* and *skp* mutants moderately reduce secretion while in a *degP* mutant translocation was abolished [8]. Interestingly, DegP acts as a chaperone and as serine endoprotease that degrades unfolded proteins. Interaction with chaperones probably prevents misfolding and keeps the PD in a loosely folded translocation-competent state [8,53] and probably prevents DegP-mediated degradation. Skp binds to the PD before translocation while SurA binds during the transit across the OM through a pore inside the β-domain [10]. The β-domain is highly conserved among SPATEs [20] and consists of a 12-stranded β-barrel, a short *N*-terminal α-helix, and a short linker loop [62] (Figure 2).

**Figure 3:**
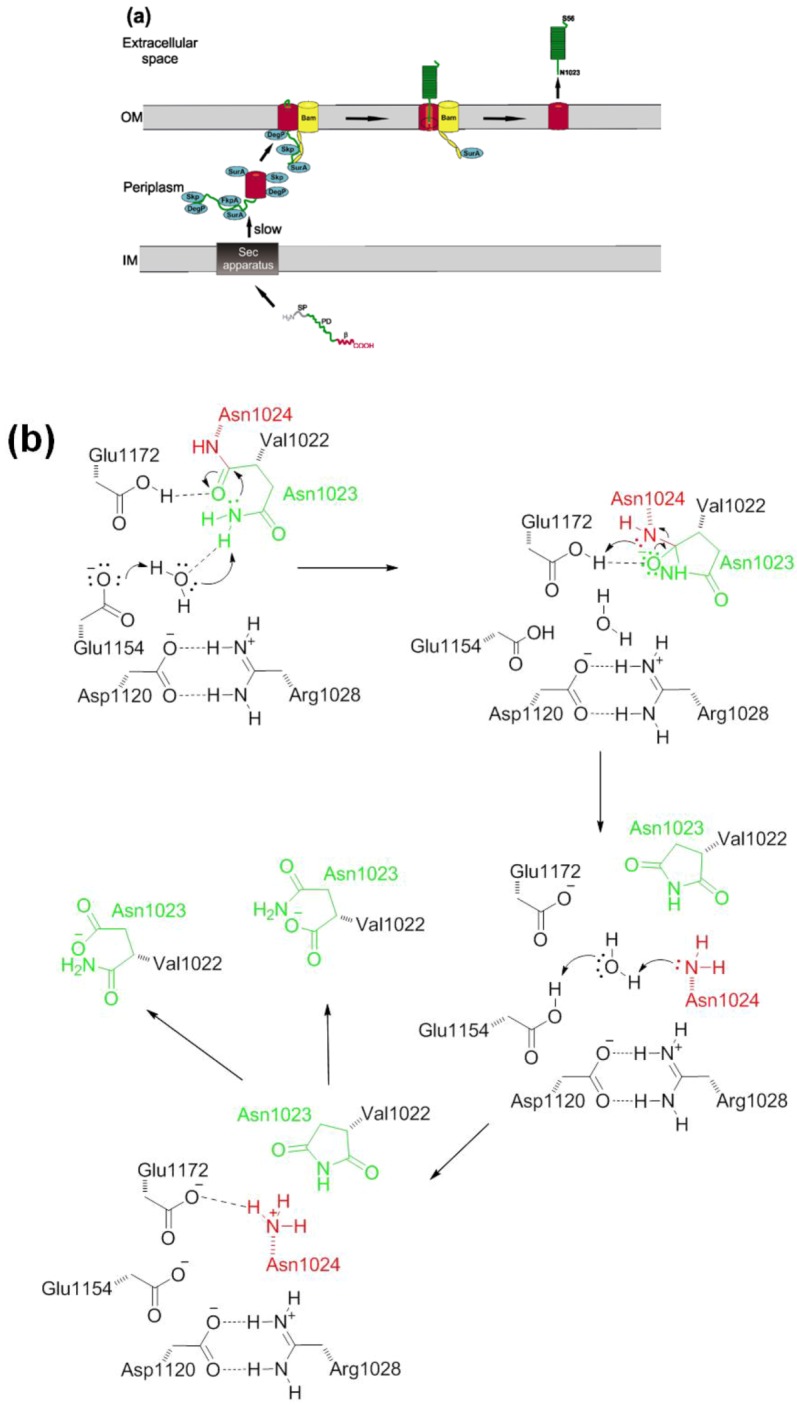
Model of EspP biogenesis. (**a**) Schematic representation of EspP secretion. EspP is translocated across the inner membrane via the sec machinery. Binding of several chaperones stabilizes EspP in a loosely-folded state in the periplasm. The β-barrel is inserted into the outer membrane by the Bam complex and other chaperones. Folding of EspP occurs in the extracellular space. When translocation across the outer membrane is complete, the passenger domain is cleaved from the β-domain autoproteolytically and transported into the extracellular space. IM, inner membrane; OM, outer membrane; SP, signal peptide; PD, passenger domain; β, β-domain; Bam, Bam complex, for clarity, only the A subunit is shown; (**b**) Proposed model of the autoproteolytic cleavage. The peptide bond between the passenger domain (Asn^1023^) and the β-domain (Asn^1024^) is cleaved inside the β-barrel. The amide group of Asn^1023^ mediates a nucleophilic attack on the peptide bond, which is catalyzed by a water molecule that forms hydrogen bonds with Glu^1154^ and other acidic residues (not shown). The intermediate oxyanion is stabilized by Glu^1172^ and results in formation of succinimide and release of Asn^1024^ as neo-*N*-terminus. The succinimide intermediate is eventually hydrolyzed to asparagine and iso-asparagine; dashed lines, hydrogen bonds [8,10,64,65].

The β-barrel domain shows considerable tertiary structure in the periplasm and during membrane integration [53], which might also be achieved by interactions with periplasmic chaperones. As with the PD, SurA, DegP [8], and Skp [10] interact with the β-domain. In addition, the Bam complex catalyzes protein assembly into the OM [7]. Ieva *et al.* suggested that Skp interacts with EspP on the periplasmic side as it translocates the IM and transfers it to SurA. SurA subsequently binds to BamA, which together with BamB and BamD interacts with the β-domain. The lipoprotein components of the Bam complex facilitate the assembly into the OM. Notably, secretion of the PD is initiated before β-domain assembly is complete [10]. The PD is translocated in a *C*- to *N*-terminal direction across the β-barrel domain [7]. When a *C*-terminal ~17 kDa segment is exposed to the cell surface folding of the PD is initiated. This traps the PD outside the cell and also provides at least part of the energy necessary for the translocation process. Folding might be the rate-limiting step in EspP biosynthesis [63]. After complete translocation of the PD to the extracellular milieu, the PD is cleaved from the β-domain within the pore of the β-barrel in the OM [10,62,64]. Passenger domains can be released from the β-domain by extracellular proteases or—as in the case of EspP—autocatalytically. EspP is cleaved between Asn^1023^ and Asn^1024^, a cleavage site that is highly conserved among SPATEs [55,64]. In the first mechanistic study, it has been suggested that the carboxyl group of Asp^1120^ activates Asn^1023^ within the β-barrel pore [64]. The Asn^1023^ amide group mediates a nucleophilic attack on the scissile bond between Asn^1023^ and Asn^1024^ thereby forming a succinimide that is further hydrolyzed into Asn and iso-asparagine via a cyclic intermediate [64] (Figure 3b). Asparagine cyclization is in general slow in peptides, underlining the catalytic mechanism of this reaction. Notably, residues important for autoproteolysis are conserved in all SPATEs suggesting a common cleavage mechanism. Barnard *et al.* demonstrated that the conserved residues E^1021^–L^1032^ (which are part of the α-helix that spans the PD-β-domain junction) constrain the active site asparagine to conformations favorable for cyclization. In addition to steric effects, positively charged residues in the α-helix interact with negatively charged residues on the barrel wall facing the lumen. This leads to formation of a salt bridge that is essential for the cleavage reaction [65]. Mutations inside the α-helix abolished autocatalytic cleavage whereas passenger translocation of EspP was not affected [66]. However, in Hbp/Tsh comparable mutations impaired both cleavage and translocation [67]. Interestingly, a recent study has demonstrated that in Hbp/Tsh the active Asn^1100^ (which is equivalent to Asn^1023^ in EspP) is not activated by the respective Asp^1197^ residue [68]. Instead, Tyr^1227^ and Glu^1249^ position a water molecule in proximity to Asn^1100^, resulting in deprotonation and activation of this residue [68]. A similar model has been proposed recently for EspP, where the nucleophilicity of the active site asparagine is enhanced by proton abstraction via water molecules that are positioned by the conserved acidic residues Asp^1120^, Glu^1154^ and Glu^1172^ [65]. The resulting oxyanion intermediate is stabilized by protonated Glu^1172^ [65]. The newly formed *N*-terminus of the β-barrel (Asn^1024^) interacts with an acidic cluster and the α-helix and the linker loop block access from the periplasmic space, which probably protects the bacterium from uncontrolled influx and efflux [62]. Whether the recently identified translocation and assembly module (TAM) that contributes to secretion of the autotransporters Ag43, EhaA, and p1121 [11] also is involved in secretion of EspP remains to be investigated. The complete secretion of EspP is illustrated in Figure 3.

### 2.3. Prevalence and Distribution of EspP

The *espP* gene has been first described on the large plasmid of O157:H7 EHEC strain EDL 933 [1] and since then, distribution and prevalence in different *E*. *coli* populations has been investigated intensively. In general, *espP* is strongly associated with STEC, EHEC and atypical enteropathogenic *E*. *coli* (EPEC) and has been detected only very rarely in other *E*. *coli* pathotypes and, to the best of our knowledge, not in commensal *E*. *coli* [18,69,70]. The *espP* gene was found in one study in 3.6% of the analyzed EAEC strains [18] and in 1.4% of typical EPEC [70]. Generally speaking, highest prevalences of *espP* have been seen in classical EHEC serotypes such as O157:H7, O26:H11/NM, or O145:H25/H28/NM [49,50,71,72,73,74]. In addition to pO157, *espP* has been described on the virulence plasmids pO113 [22], and pO26-Vir [75]. Interestingly, *espP* is not found in sorbitol-fermenting (SF)O157:NM and sequence analysis of the respective virulence plasmid has revealed that pSFO157 encodes the *sfp* fimbriae gene cluster instead of *espP* [76]. Similarly, Nagano and coworkers showed that β-glucuronidase (GUD^+^) NSF O157:H7 also do not harbor *espP* [77]. The respective large plasmid in these strains (pO157_2) also lacks *espP* [78].

Besides the variable distribution of *espP* in virulence plasmids, different *espP* alleles have been described. Brunder *et al.* first showed seven different restriction types for *espP* PCR products in a limited strain collection indicating a certain degree of heterogeneity [79]. A systematic analysis of 98 *espP*-positive EHEC and STEC strains from 56 different serotypes identified four different alleles, namely *espPα*, *espPβ*, *espPγ*, and *espPδ* [49]. The four alleles have been found in 17, 16, 15, and eight serotypes, respectively. Notably, the highly virulent serogroups O157, O26, O111, and O145 all exclusively harbored *espPα*. The encoded proteins EspPα and EspPγ are secreted (see 2.2) and proteolytically active (see 2.4) while EspPβ is either not secreted or proteolytically inactive, and EspPδ is secreted but proteolytically inactive [49], demonstrating significant functional differences of *espP* alleles on protein level. Based on the allele-specific PCR developed in this study, Khan *et al*. investigated 121 *espP*-positive strains belonging to 61 different serotypes. The serotypes O157:H7/NM, O26/H11, and O145:NM again all contained *espPα* and serotypes with lower virulence harbored *espPβ* or *espPγ*, *espPδ* has not been found in the strain collection [50]. Furthermore, *espPα* was more prevalent in human isolates (84%) than in environmental isolates (47%), while *espPγ* was more present in the environment (40%) than in humans (11%) [50], indicating that the environmental reservoir of *espP*-positive strains might differ on subtype-level. It has been suggested that further *espP* alleles might be found due to additional recombination events [49]. In accordance, Bielaszewska *et al.* described a new *espP* allel, *espPε*, that was found in one *E*. *coli* O91:H8 and 49 of 77 O91:H14/Hnt/NM [80].

Several studies have investigated the correlation of *espP* in human isolates with disease [72,74,81] and suggested that there is no significant correlation between *espP* and clinical outcome, while others found differences in prevalence of the *espP* gene in clinical isolates and environmental samples [82]. These inconsistent results underline that it is not possible to assign the virulence potential of *espP* without taking into account the functional differences of EspP subtypes. Accordingly, Khan *et al.* found the *espP* gene in 65% of isolates from patients with bloody diarrhea or HUS and in 67% of isolates from patients with watery diarrhea or without any symptoms at all. On subtype level however, all *espP*-positive strains from HUS patients harbored *espPα*, indicating a potential role of this subtype in EHEC pathogenicity [50]. It has however to be kept in mind that EHEC quite likely have developed different pathways of host injury.

Concerning the different animal and environmental reservoirs, *espP* is widely distributed and shows a certain association with cattle reservoirs. Toszeghy *et al.* found the *espP* gene in 22 of 101 bovine isolates but in none of 26 and 19 isolates of chicken and turkey, respectively [83]. Horcajo *et al.* found *espP* in 60.3% of EHEC and atypical EPEC strains in cattle but just in 11.7% of strains from sheep and goat [84]. In accordance, several other studies showed the association of the *espP* gene with bovine isolates with a prevalence of 23.8% to nearly 100% [73,81,84,85,86,87,88,89,90,91,92]. Furthermore, *espP* has been found in *E*. *coli* isolates from food samples like raw-milk, cheese [82,93], meat products [94,95], drinking water, tea [94], and ground beef [96] as well as in environmental isolates, e.g. from soil samples, ground, cattle water troughs, and feeders [50,82,97,98]. As indicated, reservoirs of *espP*-positive strains might differ on subtype-level. However most studies have only determined the presence of *espP* and not performed subtype-specific analysis.

### 2.4. Proteolytic Activity and Structure of the Passenger Domain

The passenger domain of EspP harbors a serine protease function as seen by the typical serine protease sequence motif GDSGSPLF and by the fact that it is inhibited by addition of phenylmethanesulfonylfluoride (PMSF), a specific serine protease inhibitor, but not by addition of ethylenediaminetetraacetic acid (EDTA), an inhibitor of metalloproteases [1]. The catalytic triade has been determined by site-directed mutagenesis and consists of His^127^, Asp^156^, and Ser^263^ [2].

At present, only a limited number of substrates has been identified. These are coagulation factor V, porcine pepsin A [1], apolipoprotein A-I [26], complement factor C3/C3b and C5 [99], and EHEC-hemolysin [100]. In addition, a casein-based assay has been used to determine proteolytic activity of the homologue PssA [3]. Furthermore, EspP has been shown to cleave the synthetic peptides Suc-Ala-Pro-Leu-pNA (Suc, succinic acid, pNA, para-nitroaniline) and Arg-Arg-pNA [45]. However, the latter could not be reproduced by our own studies and also no EspP substrate has been described that is cleaved *C*-terminal to Arg. Several physiological relevant proteins have been shown to resist EspP-mediated degradation namely human IgA, a human myeloma IgA1 preparation, bovine serum albumin, alpha-2-macroglobulin, transferrin, lactoferrin, pepsinogen [1], spectrin, bovine submaxillary mucin [45] as well as complement factors H and I [99].

The cleavage of complement factors has been studied in more detail. Orth *et al.* demonstrated that EspPα cleaves complement factors C3/C3b and C5 by incubation of purified proteins and human serum with EspPα and incubation of purified complement proteins with EHEC culture supernatants. Factor C3b was degraded into three major fragments with molecular masses of 42, 38, and 37 kDa, respectively, while the degradation of factor C5 into three fragments with molecular masses of 39, 37, and 33 kDa was found to be less pronounced. C5-depleted serum that was supplemented with purified C5 preincubated with EspP showed significantly reduced complement activation in all three activation pathways (classical pathway, alternative pathway, mannan-binding lectin pathway) [99]. The complement system plays a crucial role in the host response of the innate immune system and downregulation of complement activation might consequently impair host defense in EHEC infections. It has been suggested that complement downregulation might protect EspPα-secreting EHEC as well as host cells from opsonization, complement-mediated lysis, and inflammatory events [99]. Cleavage of C3 has been shown for several other bacterial proteases. These include dentilisin from *Treponema denticola* [101], interpain A from *Prevotella intermedia* [102], extracellular gelatinase from *Enterococcus faecalis* [103], streptococcal pyrogenic exotoxin B from *Streptococcus* sp. [104], as well as proteases from *Porphyromonas gingivalis* [105], and *Pseudomonas aeruginosa* [106]. Cleavage of C5 has been shown by the 56-kilodalton protease from *Serratia marcescens* [107], gingipain-R and gingipain-K from *Porphyromonas gingivalis* [108], and streptococcal C5a peptidase from *Streptococcus* sp. [109]. A potential role of EspPα is underlined by the finding that specific antibodies have been isolated from sera of HUS patients [1,3].

Another potentially relevant substrate *in vivo* might be coagulation factor V (FV), which is present in plasma and in α-granules of platelets. FV plays a role in hemostasis and can act either as procoagulant or as anticoagulant (reviewed in [110]). Brunder *et al.* have suggested that this degradation could lead to decreased coagulation possibly leading to prolonged bleeding and increased hemorrhage in the gastro intestinal tract that can be observed during EHEC infection [1]. Together with further virulence factors such as EHEC-Hly [111] this might lead to improved supply of iron via hemoglobin. The ability to degrade FV is widely distributed within the SPATE family. Pet and Pic from EAEC, Sat from UPEC, and EspC from EPEC also cleave this protein. Also, Tsh from avian pathogenic *E*. *coli* was found to degrade FV when a purified protein was used but not in human plasma [45]. Unfortunately, functional investigations have not been performed so far.

Apolipoprotein A-I (apo A-I) has been reported to be cleaved by EspPα and EspI, another SPATE, which is also found in EHEC [26]. However, degradation has not been investigated on a functional level. Apo A-I is known to be involved in lipid binding and transport [112]. More interestingly, Concha *et al.* suggested that during bacterial infection apo A-I could release antimicrobial peptides from HDL (high density lipoprotein) particles after proteolysis [113]. Furthermore, apo A-I is a negative acute phase protein with anti-inflammatory properties [114] that can directly bind lipopolysaccharides (LPS) [115] and is capable of inactivating endotoxins and decreasing LPS-induced cytokine release [116,117]. Therefore, degradation of apo A-I by EspPα might either activate or inhibit its antimicrobial function and could interfere with inflammatory processes during EHEC infection. Again, experimental data in model systems concerning the functional implications of apo A-I cleavage are missing.

In addition, it has been demonstrated that EspPα cleaves EHEC hemolysin (EHEC-Hly) in its free and vesicle-bound form [100]. EHEC-Hly belongs to the repeat-in-toxin (RTX) family [118] and is able to lyse erythrocytes and lymphocytes [118,119] and injures microvascular endothelial cells [120]. Functional characterization was performed using purified proteins, supplementation of purified EspPα to culture supernatants of EHEC-Hly expressing strains, and coexpression of both proteins [100]. Cleavage of EHEC-Hly occurs in the hydrophobic domain which is important for the cytolytic activity of RTX toxins [121] and consequently leads to loss of hemolytic activity. In a cellular infection model using human brain microvascular endothelial cells (HBMEC), coexpression of EspPα and EHEC-Hly in recombinant *E*. *coli* resulted in basal HBMEC cytolysis whereas pronounced dose-dependent cytolysis was observed in controls [100]. It has been proposed that pathogenic *E*. *coli* might be able to regulate their virulence phenotypes by interference of effector molecules [100]. While cleavage and inactivation has been, to the best of our knowledge, only described so far for EspP and EHEC-Hly, other RTX toxins are activated by proteolytic cleavage. For example, various proteases of *Vibrio cholerae* cleave and activate the El Tor cytolysin/hemolysin [122]. Another example is the proteolytic activation of Shiga toxins by eukaryotic proteases [123,124].

**Figure 4:**
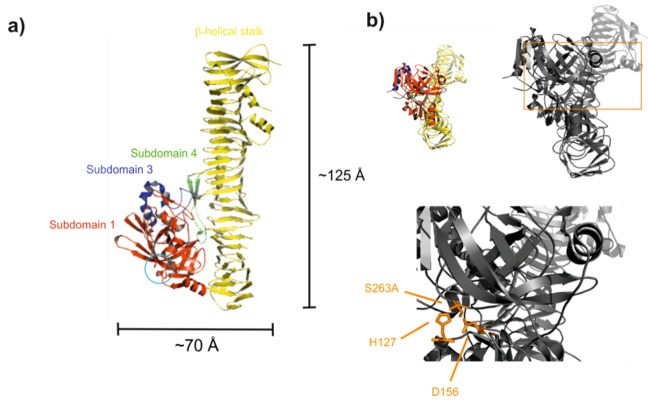
EspPα passenger domain. (**a**) Ribbon diagram of the EspPα passenger domain. EspPα is composed of a β-helical stalk and three globular subdomains (SD), red, SD 1 (residues 56–313), blue, SD 3 (residues 596–630), green, SD 4 (residues 671–699), yellow, β-helical stalk. In contrast to Hbp and IgAP, EspPα does not exhibit the large SD 2 protruding from the β-helical stalk. Instead, SD 3 in EspPα is much larger than in Hbp and IgAP and shows similarity to a domain termed 2A in Pet [128]. SD 3 exhibits a disordered loop containing the only two cysteine residues in the entire passenger. The blue circle indicates the position of the catalytic triad; (**b**) Catalytic triad of EspPα. Top: Overview of EspPα. Localization of the detailed view (bottom) is highlighted by the orange rectangle. Bottom: Residues of the catalytic triad (H^127^, D^156^, and S^263^) are shown as orange sticks. Ser^263^ is exchanged by Ala in the crystal structure [125].

Khan *et al.* solved the 2.5 Å crystal structure of the EspP passenger domain using the proteolytically inactive mutant S263A [125]. It consists of a large β-helical stalk and a globular subdomain that harbors the catalytic triad (see Figure 4). The overall fold of EspP is typical for known autotransporters and resembles that of *E*. *coli* hemoglobin protease (Hbp) [126] and *Haemophilus influenza* immunoglobulin A protease (IgAP) [127].

The globular subdomain shows similarity to the chymotrypsin family. However, the S1 subsite of EspPα is narrower and shallower as well as slightly more hydrophobic than that of bovine chymotrypsin. Therefore, Khan *et al.* suggested that EspPα cleaves after smaller and more hydrophobic amino acids than chymotrypsin, which cleaves after tyrosine, tryptophane, phenylalanine, leucine, and methionine. This suggestion is in good accordance with the cleavage sites that have been determined so far where EspPα mainly cleaves after Leu [1,45,99,100]. When compared to Hbp and IgAP, the active site cleft of EspPα is slightly wider and much deeper indicating different substrate specificities. Furthermore, it is more exposed than that of Hbp and much more exposed than that of IgAP, leading to the suggestion that EspPα interacts with a larger surface of substrates or with larger substrates in general [125]. This is in accordance with the finding that EspPα is able to cleave porcine pepsin A but not its precursor pepsinogen [1] suggesting that not only the amino acid sequence but also the three-dimensional structure might be important for substrate recognition. This might be amongst other effects mediated by the β-helical stalk as proposed by Khan *et al.* [125]. 

Taken together, EspPα seemingly shows high substrate specificity. There are only a few known human substrates, which are related to blood coagulation and immune response. Cleavage of these substrates might contribute to the severity of EHEC infections. Also, EspP might be involved in regulation of *E*. *coli* virulence as shown by cleavage of EHEC hemolysin. Cleavage predominantly occurs after leucine at position P1 and other small hydrophobic amino acids at position P2.

### 2.5. Cytotoxicity and Cellular Adherence

Cytotoxicity of EspP is discussed controversially. Incubation of Vero cells with culture supernatants of PssA secreting *E*. *coli* strains resulted in cytotoxic effects as analyzed by fluorescence microscopy with phalloidin staining. Dependent on incubation time the cells showed defects in cell-cell junctions, retraction of cell bodies, and loss of stress fibers (5 h), with disruption of the actin cytoskeleton in about 30% of cells, loss of cell-cell junctions, detachment from the substratum (10 h), and cell death at later time points [3]. It was suggested that these effects might be due to apoptosis, however cytotoxic pathways have not been analyzed. In contrast, Dutta *et al*. were not able to reproduce any cytopathic effects on Vero cells after incubation with 0.5 and 1 μM EspP for 5–30 h. In addition, no cytotoxic effects have been shown for the epithelial cell line HT-29 and HEp-2 in this study [45]. In our own studies, we neither found any cytotoxic effects in epithelial HT-29 cells nor in endothelial HBMEC after incubation for 30 h or 48 h with up to 1 μM EspPα (Brockmeyer, unpublished data). A recent study used the epithelial HeLa cells and observed binding of EspPα after 6 h of incubation in a dose-dependent manner [129]. Prolonged incubation (24 h) with EspPα at higher concentrations lead to cell rounding in this study. Since EspPα did not induce LDH release, Xicohtencatl-Cortes *et al*. stated that it showed cytopathic but not cytotoxic effects. Again, potential underlying mechanisms have not been analyzed.

Initial motivation to analyze the potential of EspPα to contribute to cellular adherence or biofilm formation was raised by the finding that pO157 is required for full adherence of *E*. *coli* to Henle 407 intestinal and HEp-2 epithelial cells [130] and influences the colonization of the bovine terminal rectum [131]. In addition, pO157 is important for biofilm formation and adherence to the epithelial T84 cells [132]. Puttamreddy *et al.* generated a transposon insertion library in *E*. *coli* strain EDL933 and identified 51 distinct genes or intergenic regions responsible for biofilm formation. Among the genes responsible for full biofilm potential is *espP*α which also contributed to adherence of T84 cells [133]. 

A potential mechanism for biofilm formation and adherence mediated by EspPα has been published recently by Xicohtencatl-Cortes *et al*. [129]. Under certain conditions, EspPα is able to form macroscopic rope-like structures via oligomerization. These ropes are up to 2 cm in length, and show pronounced stability against mechanical stress, high temperature, and detergents [129]. Under laboratory conditions, the ropes formed a substratum for bacterial biofilm formation and were able to bind exogenously added bacteria [129]. Bacteria associated with the ropes were protected from antimicrobial drugs, indicating a potential protective role for the bacterial population within ropes. Like monomeric EspPα, ropes also bind to HeLa cells [129]. Whether the rope-like structures are formed during infection is still elusive.

### 2.6. Gene Expression

Studies regarding *espP* gene expression are rare and mostly descriptive. Ebel *et al*. observed enhanced EspP levels in EHEC culture supernatants when bacteria were grown in lysogeny broth (LB) medium rather than in minimal essential medium (MEM), indicating that a medium rich in amino acids leads to higher EspP production [134]. In addition, more EspP is produced at 37 °C than at 20 °C [1,134] and pH 7 and pH 9 are favorable compared to pH 5 at which EspP production is nearly completely abolished [1]. Based on the methodology used by Ebel *et al.* and Brunder *et al.* it is not possible to differentiate between effects on gene expression or protein secretion. Enhanced secretion under slightly alkaline conditions indicates that EspP might act on the human colon or in the circulation. Indeed, we have recently shown a potential interaction of EspP with the human intestine [100]. Incubation of EHEC in contact with human intestinal epithelial HCT-8 cells lead to upregulation of *espP* expression up to more than 35-fold [100].

## 3. Conclusions

EHEC are pathogens that cause severe diseases in humans worldwide. Their pathogenicity is a multifactorial process and besides the well-known Shiga toxins, EHEC express a variety of further virulence factors. EspP belongs to the SPATE family and has been subject of many studies concerning the autotransport mechanism. Thus, its biogenesis has been investigated extensively. The espP gene is highly prevalent in EHEC and to date five EspP subtypes are known that differ in their proteolytic activity and in their ability to translocate across the bacterial cell membranes. Therefore, mere detection of the espP structural gene without subtyping is not sufficient to investigate a potential correlation with disease as claimed by some authors. Studies addressing the distribution of EspP subtypes indicate that the proteolytic active and translocation-competent subtype EspPα is associated with highly pathogenic EHEC serotypes including O157:H7 as well as with isolates from patients with severe disease. Hence, nearly all recent studies have been performed (although not always termed using subtype nomenclature) with EspPα. This subtype might contribute to biofilm formation possibly by the formation of rope-like structures that in addition might protect the bacteria inside these structures from host defense.

As suggested for SPATEs in general, the virulence of EspPα might be mediated by its proteolytic activity. Based on the currently available data, EspPα shows pronounced specificity. Notably, EspPα is able to cleave another EHEC virulence factor, namely EHEC-Hly. This abolishes the hemolytic and cytolytic activity of EHEC-Hly and leads to the suggestion that EHEC bacteria might be able to self-control their virulence phenotype by effector molecule interference. In addition, different host proteins are degraded by EspPα, e.g. complement factors C3 and C5 as well as coagulation factor V. Cleavage of the complement factors significantly reduces complement activation in all activation pathways possibly resulting in diminished host response and increased the severity of EHEC infections. Degradation of coagulation factor V has been suggested to lead to prolonged bleeding and increased hemorrhages during EHEC infection. Functional consequences of FV cleavage have, however, not been addressed yet.

Based on structural homology and functional similarity, SPATEs have been divided into two groups, the cytotoxic class I SPATEs and the non-cytotoxic class II SPATEs [45]. EspP has been classified as class I SPATE. Its cytotoxicity is however discussed controversially. The further class I SPATEs, Sat, Pet, SigA and EspC, have all been shown to degrade spectrin/fodrin [24,35,45,135] after internalization of the host cell and it has been demonstrated that this activity underlies cytotoxicity [23,34,41,45] although details in cytotoxic mechanism differ [133,134]. In contrast, EspP, like all class II SPATE, does not cleave spectrin/fodrin [44] and available data on cytotoxicity range from lack of cytopathic effects to pronounced cytotoxicity. Notably, the potential cytotoxic mechanism (which might also explain the controversial results) has not been investigated yet and no data are available in support of cellular internalization of EspP. Together, EspP can be regarded as a “functional outlier” in the class I SPATE group.

Coagulation factor V, is also not exclusively cleaved by EspP but in addition also by class I SPATEs EspC, Pet, Sat, and the class II SPATEs Pic, and Tsh/Hbp [45]. A further substrate of EspPα, pepsin, is cleaved by EpeA, EspC, EspI, Pet, and Pic [22,26,35,45]. Several class II SPATEs show mucinolytic activity (EpeA, Pic, and Tsh/Hbp) which is not seen for class I SPATEs including EspP [22,35,45]. Interestingly, a recent study provided evidence that Pic degrades various leukocyte glycoproteins resulting in impaired chemotaxis and migration. In addition, Tsh/Hbp showed a similar substrate spectrum, indicating that class I SPATE might interfere with host response [135]. Although via a different pathway, EspP might also affect host response via degradation and inactivation of complement factors. 

Together, EspP has been shown to mediate different functions ranging from biofilm formation to subversion of host defense. The functional properties of this SPATE are in between the *bona fide* known class I SPATE (cytotoxic via spectrin degradation, no mucinolytic activity) and class II SPATE (not cytotoxic, no spectrin degradation, mucinolytic activity, probably interference with host defense). It has to be noted that almost all studies have been performed *in vitro* and their relevance *in vivo* has not been demonstrated yet due to the lack of suitable animal models. Nevertheless, future studies will help to understand the function and characteristics of EspP in more detail to further elucidate its role as a virulence factor during EHEC infection. 
